# Toll-Like Receptor 3/TRIF-Dependent IL-12p70 Secretion Mediated by *Streptococcus pneumoniae* RNA and Its Priming by Influenza A Virus Coinfection in Human Dendritic Cells

**DOI:** 10.1128/mBio.00168-16

**Published:** 2016-03-08

**Authors:** Laura Spelmink, Vicky Sender, Karina Hentrich, Thomas Kuri, Laura Plant, Birgitta Henriques-Normark

**Affiliations:** a Department of Microbiology, Tumor and Cell Biology, Karolinska Institutet, Stockholm, Sweden; b Department of Clinical Microbiology, Karolinska University Hospital, Stockholm, Sweden

## Abstract

A functional immune response is crucial to prevent and limit infections with *Streptococcus pneumoniae*. Dendritic cells (DCs) play a central role in orchestrating the adaptive and innate immune responses by communicating with other cell types via antigen presentation and secretion of cytokines. In this study, we set out to understand how pneumococci activate human monocyte-derived DCs to produce interleukin-12 (IL-12) p70, an important cytokine during pneumococcal infections. We show that IL-12p70 production requires uptake of bacteria as well as the presence of the adaptor molecule TRIF, which is known to transfer signals of Toll-like receptor 3 (TLR3) or TLR4 from the endosome into the cell. While TLR4 is redundant for IL-12p70 production in DCs, we found that TLR3 is required to induce full IL-12p70 secretion. Influenza A virus (IAV) infection of DCs did not induce IL-12p70 but markedly upregulated TLR3 expression that during coinfection with *S. pneumoniae* significantly enhanced IL-12p70 secretion. Finally, we show that pneumococcal RNA can act as a bacterial stimulus for TLR3 and that it is a key signal to induce IL-12p70 production during challenge of DCs with pneumococci.

## IMPORTANCE

*Streptococcus pneumoniae*, a common colonizer of the nose, is the causative agent of severe and deadly diseases. A well-orchestrated immune response is vital to prevent and limit these diseases. Dendritic cells (DCs) reside in the mucosal linings of the lungs and sample antigens. They are activated by pathogens to present antigens and secrete cytokines. While many studies focus on murine models, we focused our work on human monocyte-derived DCs. We found that pneumococcal RNA is an important stimulus in DCs to activate the endosomal receptor TLR3, a receptor previously not identified to sense pneumococci, and its adaptor molecule TRIF. This leads to secretion of the cytokine interleukin-12 (IL-12). Severe pneumococcal pneumonia occurs closely after influenza A virus (IAV) infection. We show that IAV infection upregulates TLR3 in DCs, which sensitizes the cells to endosomal pneumococcal RNA. This new insight contributes to unlock the interplay between pneumococci, IAV, and humans.

## INTRODUCTION

*Streptococcus pneumoniae* is a common colonizer of the upper respiratory tract, with the potential to cause mild diseases, like otitis media and sinusitis, or life-threatening diseases, such as pneumonia, sepsis, or meningitis. It is estimated by the WHO that more than 800,000 children under the age of 5 years die annually as a consequence of pneumococcal infection (1). The main targets of pneumococcal diseases are young children (1) and people over the age of 65 years (2), immunocompromised individuals, and people infected with HIV (3). Severe and deadly pneumococcal pneumonia also occurs in close temporal proximity after influenza A virus (IAV) infection (4, 5). This has been observed during IAV pandemics (6), as well as during seasonal outbreaks (7).

The immune state of the host is a key factor determining the outcome of pneumococcal infections. The first line of defense against a pneumococcal encounter in the respiratory tract is the innate immune response. Pattern recognition receptors (PRRs), such as the membrane-bound Toll-like receptors (TLRs) and cytosolic NOD-like receptors (NLRs), play an important role in innate detection of pneumococci. Several pneumococcal components have been implicated in the activation of NLRs and TLRs (reviewed in reference 8). Pneumococcal peptidoglycan has been shown to activate NOD2 (9, 10), the Gram-positive cell wall component lipoteichoic acid (LTA) activates TLR2 (11), and the pore-forming toxin pneumolysin has been reported to activate TLR4 (12–16). We previously identified a nonredundant role of TLR9 *in vivo* (17) and a central role of the adaptor molecule MyD88 in controlling pneumococcal colonization and systemic spread (18). While MyD88 acts as an adaptor for several TLRs, the adaptor molecule TRIF only mediates signal transmission from TLR4 and TLR3 into the cell (19, 20).

DCs are a central part of the immune response, because they link innate and adaptive immunity. They are located in the mucosal linings of the lungs and constantly sample antigens. Upon encounter with a pathogen, PRRs are activated and induce the DCs to present large amounts of antigen on their surface and to produce proinflammatory cytokines. DCs are the main producers of interleukin-12 (IL-12), an important proinflammatory cytokine which drives the differentiation of T_H_1 cells and induces other innate immune cells to produce cytokines such as gamma interferon (IFN-γ). These responses are common in infections with intracellular pathogens, but they are also found in infections with the extracellular pathogen *S. pneumoniae* (21–23). IL-12p40-deficient mice show decreased IFN-γ production, neutrophil recruitment, and survival in a pneumococcal pneumonia model (21) which can be reversed following administration of exogenous IL-12 (21, 24). It has also been reported that a patient with a severe deficiency in IL-12 production suffered from recurrent pneumococcal infections (25), which underlines the importance of IL-12 in the immune response to pneumococci.

IAV infection affects the host in multiple ways that contribute to the severe outcome of secondary pneumococcal infections (reviewed in reference 26). The effects include systemic immunosuppression (27), the modulation of cytokine responses to pneumococci (28, 29), and changes in the expression of and exposure to pneumococcal receptors (30, 31). The cytokines IL-12p70, IL-6, and IL-15 have been identified in a patient study as markers for severe disease outcome after IAV infection (32). Additionally, *in vitro* studies have shown that IAV infection of human monocyte-derived DCs triggers an enhanced secretion of IL-12p70, IL-6, tumor necrosis factor alpha, and IFN-γ in response to secondary pneumococcal infection (33, 34).

Murine models have been essential to advance our knowledge about IAV and pneumococcal infections, but they also have limitations for studies of human pathogens. Human and murine DCs differ in their potential to produce IL-12p70 and IL-1β in response to pneumococci (35), and therefore we used an *in vitro* model of human monocyte-derived DCs to study their role in the context of pneumococcal infections and coinfection with IAV.

We studied the TRIF-dependent signaling in DCs challenged with pneumococci and in coinfections with IAV, and we found a TRIF dependency for IL-12 expression and secretion. Surprisingly, IL-12p70 production is not mediated by TLR4 but depends on the double-stranded RNA (dsRNA) receptor TLR3. Furthermore, we show that pneumococcal RNA activates TLR3 and that it is a sufficient and required stimulus for IL-12p70 production in DCs.

## RESULTS

### IL-12p70 production by DCs requires internalization of pneumococci into the endosomal compartment and the adaptor molecule TRIF.

Cytokine induction was investigated after infection of human DCs with the T4R strain. The nonencapsulated strain T4R was used as an alternative to opsonization of the encapsulated wild-type T4 strain, since it was shown previously that both induce comparable amounts of uptake and cytokine induction in DCs (35). Hence, by using T4R we could avoid introducing a higher degree of complexity into our model. At a low multiplicity of infection (MOI) of 1, we did not observe cytotoxic effects on DCs (see Fig. S1A in the supplemental material). Additionally, IL-12p70 secretion was abolished in DCs treated with the uptake inhibitors cytochalasin D and wortmannin, indicating that pneumococci activate an intracellular receptor to induce cytokine responses (see Fig. S1B in the supplemental material).

**FIG 1:**
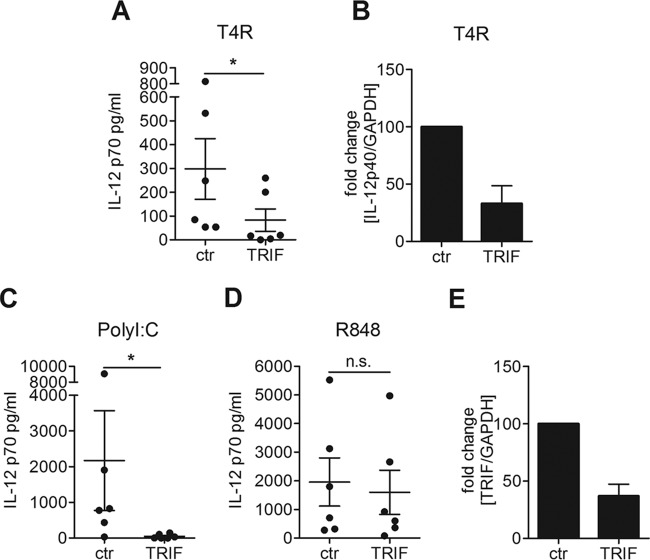
IL-12p70 production by DCs challenged with pneumococci requires the adaptor molecule TRIF. DCs were treated with siRNA against TRIF or random control siRNA. The cells were infected with T4R (A and B) or stimulated with PolyI:C (C) or R848 (D) to confirm the knockdown. IL-12p70 secretion was measured in the cell culture supernatant by ELISA; each dot represents the result from one donor (A, C, and D). Fold changes in gene expression of IL-12p40 or TRIF relative to control cells were measured using RT-PCR (B and E). Graphs show means ± standard errors of the means for results from 6 (A, C, and D), 2 (B), or 4 (E) experiments. Statistical analysis was performed using a Wilcoxon matched-pairs signed-rank test (for ELISAs) or Student’s *t* test (for RT-PCRs). *, *P* < 0.05. n.s., not significant.

TRIF is an important adaptor molecule for signaling from the endosomal compartment, since it mediates the signals from TLR3 and TLR4 into the cell. We studied the impact of TRIF on IL-12p70 production by silencing TRIF in DCs via small interfering RNA (siRNA). Upon stimulation with T4R, we found that IL-12p70 production was significantly reduced by the TRIF knockdown (Fig. 1A). The TRIF dependency was also confirmed by studying the expression of IL-12p40 using real time-PCR (RT-PCR) (Fig. 1B). The knockdown was confirmed functionally by stimulating DCs with PolyI:C (a TLR3 agonist) or R848 (a TLR7/8 agonist), and RT-PCR analysis showed a 60% knockdown of the TRIF transcript (Fig. 1C to E). Our results demonstrated an important role of the endosomal adaptor molecule TRIF for IL-12 production in DCs challenged with T4R.

### IL-12p70 production in DCs in response to pneumococci depends on TLR3 but not TLR4.

**FIG 2:**
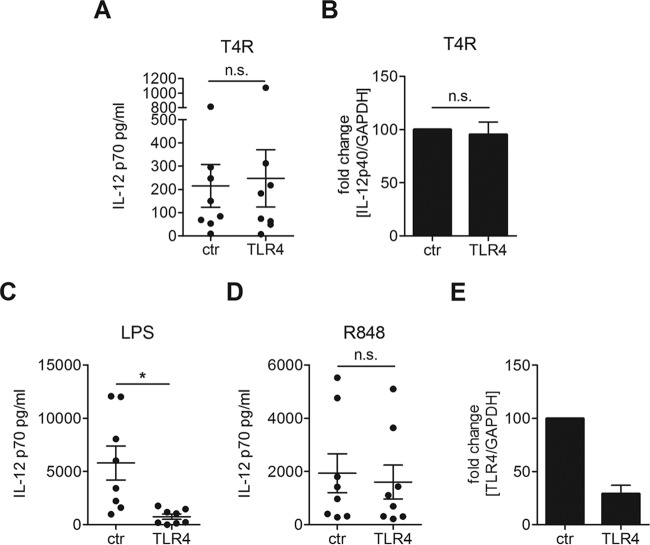
DCs challenged with pneumococci produce IL-12p70 independently of TLR4. DCs were treated with siRNA against TLR4 or random control siRNA. The cells were infected with T4R (A and B) or stimulated with LPS (C) or R848 (D) to confirm the knockdown. IL-12p70 secretion was measured in the cell culture supernatant by ELISA; each dot represents the results from one donor (A, C, and D). Fold changes in gene expression of IL-12p40 or TLR4 relative to control cells were measured using RT-PCR (B and E). Graphs show means ± standard errors of the means of results for 7 (A, C, and D), 3 (B), or 5 (E) experiments. Statistical analysis was performed using a Wilcoxon matched-pairs signed-rank test (for ELISAs) or Student’s *t* test (for RT-PCRs). *, *P* < 0.05. n.s., not significant.

Since TRIF mediates the signals from both TLR3 and TLR4 into the cell and pneumolysin has been suggested to activate TLR4, we first set out to investigate whether TLR4 is activated by T4R in our infection model. We silenced TLR4 with siRNA in DCs and found that IL-12p70 production in response to T4R was independent of TLR4 (Fig. 2A). Similarly, the expression of IL-12p40 did not require TLR4 (Fig. 2B). The knockdown was confirmed functionally by stimulating with lipopolysaccharide (LPS, a TLR4 agonist) or R848, and RT-PCR analysis indicated that 70% of the RNA transcript was silenced (Fig. 2C to E). These data show that IL-12p70 production in DCs does not require the receptor TLR4.

**FIG 3:**
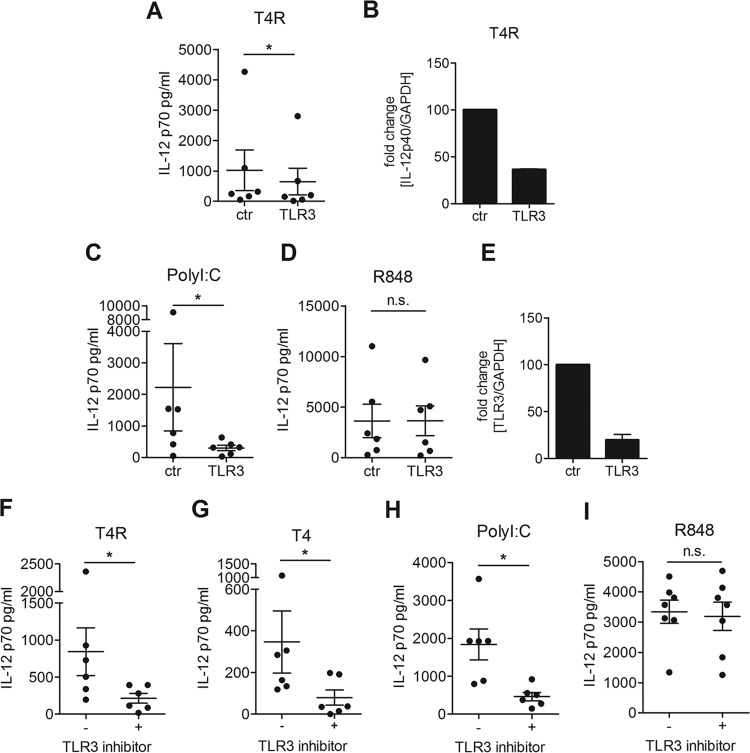
TLR3 is required for IL-12p70 production by DCs challenged with pneumococci. DCs were treated with siRNA against TLR3 or random control siRNA (A to E) or treated with a TLR3/dsRNA complex inhibitor (F to I). The cells were infected with T4R (A, B, and F), opsonized T4 (G), or stimulated with PolyI:C (C and H) or R848 (D and I) to confirm the knockdown. IL-12p70 secretion was measured in the cell culture supernatant by ELISA; each dot represents the results from one donor (A, C, D, and F to I). Fold changes in gene expression of IL-12p40 or TLR3 relative to control cells were measured using RT-PCR (B and E). Graphs show the means ± standard errors of the means of results for 6 (A, C, D, and F to I), 2 (B), or 3 (E) experiments. Statistical analysis was performed using a Wilcoxon matched-pairs signed-rank test (for ELISAs) or Student’s *t* test (for RT-PCRs). *, *P* < 0.05. n.s., not significant.

Since IL-12p70 production was independent of TLR4 but required TRIF, we next tested whether pneumococci are recognized by TLR3, an endosomal receptor shown to recognize dsRNA but that has not previously been identified as a receptor for pneumococci. Upon silencing of TLR3 with siRNA, a significant reduction in IL-12p70 production was observed in DCs challenged with T4R (Fig. 3A). In accordance with this finding, the expression of IL-12p40 was reduced by the TLR3 knockdown (Fig. 3B). The knockdown was confirmed functionally by stimulating DCs with PolyI:C or R848, and RT-PCR analysis showed that 80% of the transcript was silenced (Fig. 3C to E). To confirm the TLR3 dependency observed in our siRNA studies, we treated DCs with a TLR3/dsRNA complex inhibitor and found a strong reduction in IL-12p70 in response to T4R (Fig. 3F). Additionally, we opsonized the encapsulated strain T4 and tested whether the capsule influences the recognition of pneumococci by TLR3. We found that the IL-12p70 secretion by DCs infected with T4 could be inhibited by the TLR3/dsRNA complex inhibitor in a similar manner as T4R-infected DCs (Fig. 3G). The inhibitor also reduced IL-12p70 production in DCs stimulated with PolyI:C but not in R848-stimulated cells, confirming the specificity of the inhibitor (Fig. 3H and I). Our data showed that pneumococci can be recognized by DCs via TLR3 and its adaptor TRIF and that the TLR3/TRIF axis is important for IL-12p70 production.

### Upregulation of TLR3 by IAV leads to increased IL-12p70 production in IAV-pneumococcus coinfections.

**FIG 4:**
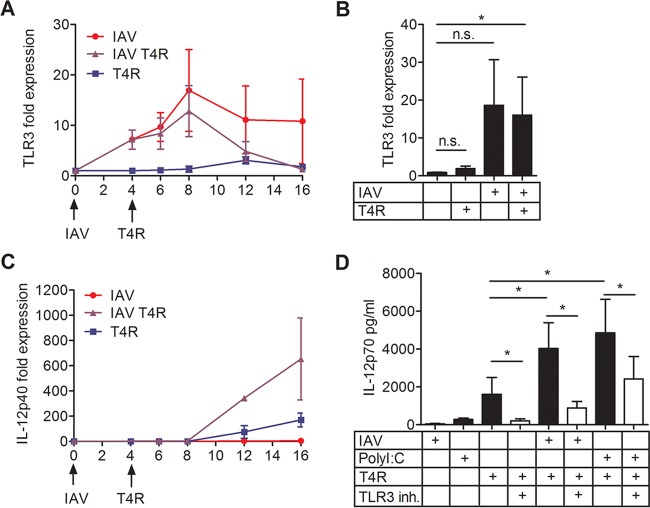
The role of TLR3 in IAV-pneumococcal coinfection. DCs were infected with IAV and/or T4R. Expression of TLR3 (A and B) or IL-12p40 (C) was measured by RT-PCR at the indicated time points (A and C) or after 8 h (B). DCs were treated with IAV, PolyI:C, or T4R, with or without the TLR3/dsRNA complex inhibitor, as indicated. IL-12p70 secretion was measured in the supernatant by ELISA (D). Values represent means ± standard errors of the means for results from 3 (A), 4 (B), 2 (C), or 6 (D) experiments. Statistical analysis was performed using Student’s *t* test (for RT-PCRs) or the Wilcoxon matched-pairs signed-rank test (for ELISAs). *, *P* < 0.05. n.s., not significant.

We previously described a type I IFN-dependent cytokine boost in IAV-pneumococcus coinfections. IAV triggers type I IFN production in infected DCs, which in turn primes the surrounding DCs to secrete increased amounts of IL-12p70 (33). We set out to investigate whether this cytokine boost is caused by an IAV-triggered upregulation of TLR3 expression. We found an increase in TLR3 expression, which peaked at 8 h post-IAV infection, both in IAV singly infected DCs as well as in DCs first infected with IAV and then 4 h later with T4R (Fig. 4A and B). T4R single infection had no effect on TLR3 expression (Fig. 4A and B). IAV single infection had no effect on IL-12p40 expression, in contrast to coinfection with T4R, where the IL-12p40 level started to increase 4 h after T4R was added to the DCs (Fig. 4C). IL-12p40 expression in T4R singly infected DCs also increased after 4 h, but at a lower level (Fig. 4C). To test whether type I IFNs caused the increased TLR3 expression in coinfected DCs, we also measured TLR3 expression in DCs treated with IFN-α after 8 h. We found only a small increase in the expression of TLR3, which does not fully explain the observed effects of IAV infection on TLR3 expression (see Fig. S2A in the supplemental material). The boosted IL-12p70 secretion after coinfection with IAV and T4R was inhibited by addition of the TLR3/dsRNA complex inhibitor, and similar results were obtained when the TLR3 agonist PolyI:C was used as a primary stimulus (Fig. 4D). Additionally, IFN-α as the primary stimulus enhanced the IL-12p70 production, and this boost was significantly inhibited by the TLR3/dsRNA complex inhibitor (see Fig. S2B in the supplemental material).

### Pneumococcal RNA is a sufficient stimulus to induce TLR3 activation and IL-12p70 production in human DCs.

**FIG 5:**
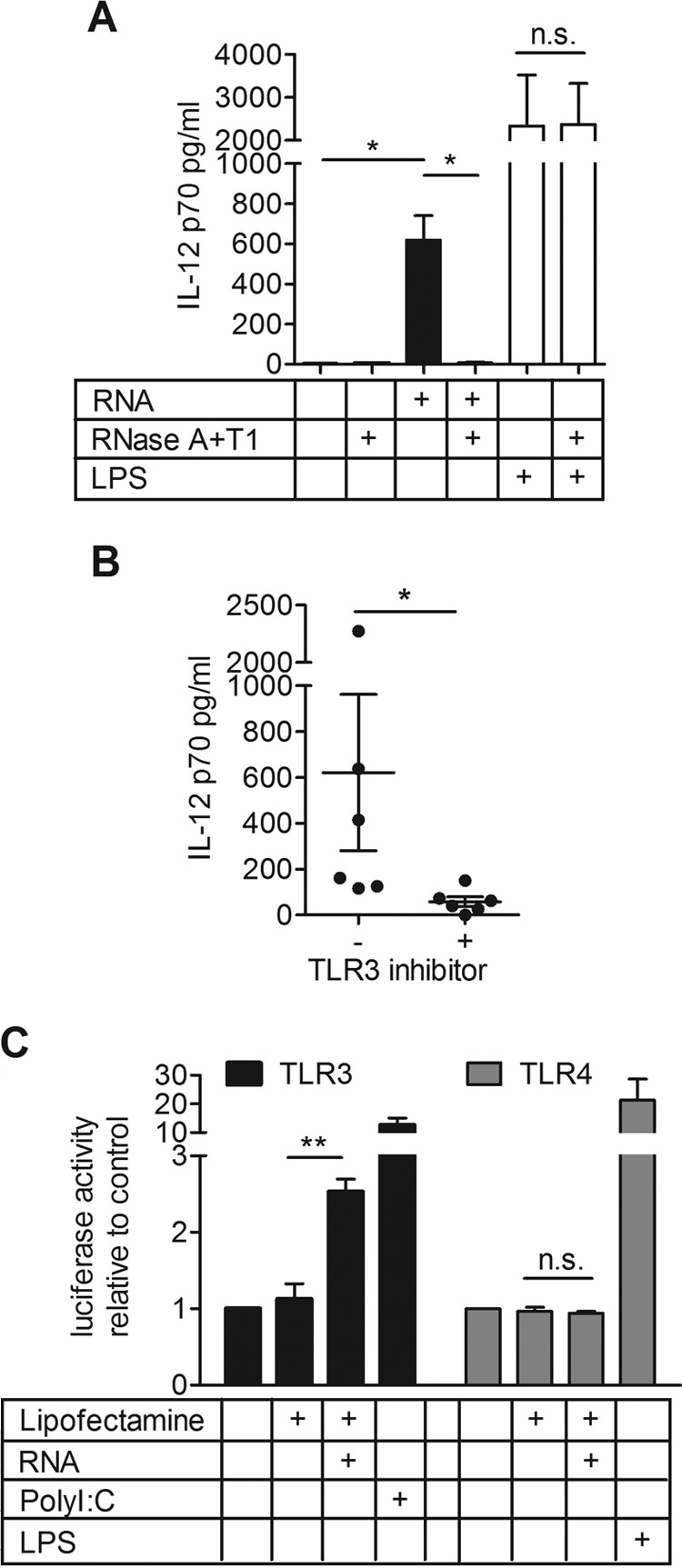
Purified pneumococcal RNA is sufficient to induce IL-12p70 in DCs and can activate TLR3. DCs were transfected with total RNA from T4R with or without prior digestion with RNase A and T1, or transfected with the enzymes alone, and a proportion of these cells were treated with LPS 4 h posttransfection (A). DCs were transfected with total RNA from T4R and treated with a TLR3/dsRNA complex inhibitor (B). IL-12p70 production was measured in the supernatant by ELISA (A and B). HEK293 cells expressing TLR3 or TLR4 were transfected with total RNA from T4R, stimulated with PolyI:C or LPS, and luciferase activity was measured (C). Values represent means ± standard errors of the means for results from 6 (A and B) or 3 (C) experiments. IL-12p70 secretion was measured in the cell supernatant. Statistical analysis was performed using a Wilcoxon matched-pairs signed-rank test (for ELISAs) or using Student’s *t* test (for the luciferase assay). *, *P* < 0.05; **, *P* < 0.005. n.s., not significant.

To understand whether pneumococcal RNA alone is sufficient to induce IL-12p70 production, total pneumococcal RNA from T4R or RNA digested with a cocktail of the endonucleases RNase A and RNase T1 was transfected into DCs. In combination, RNases A and T1 cleave RNA behind the C, G, and U residues, which results in nearly complete degradation of RNA. Total pneumococcal RNA was indeed a sufficient stimulus to induce IL-12p70 production in DCs, and RNA digested with the cocktail of RNases A and T1 lost its ability to activate DCs, indicating that there were no other components in our RNA preparation that activate the cells (Fig. 5A). To determine whether total RNA activates the same pathway as whole bacteria, DCs were transfected with pneumococcal RNA and also treated with the TLR3/dsRNA complex inhibitor. Stimulation of IL-12p70 secretion by transfected pneumococcal RNA was found to be TLR3 dependent (Fig. 5B). Additionally, HEK293 cells expressing a luciferase reporter system as well as TLR3 or TLR4 were transfected with pneumococcal RNA. A significant increase in luciferase activation was observed in TLR3-expressing, but not in TLR4-expressing, HEK293 cells (Fig. 5C). Furthermore, we used IAV as a primary stimulus and total RNA from T4R as the secondary stimulus, but we could only observe a nonsignificant trend to increased IL-12p70 production relative to bacterial RNA alone (see Fig. S2C in the supplemental material).

Our results showed that pneumococcal RNA can induce production of IL-12p70 by DCs and that pneumococcal RNA can act as a stimulus for TLR3.

### Pneumococcus-induced IL-12p70 production by DCs requires RNA as a signal.

**FIG 6:**
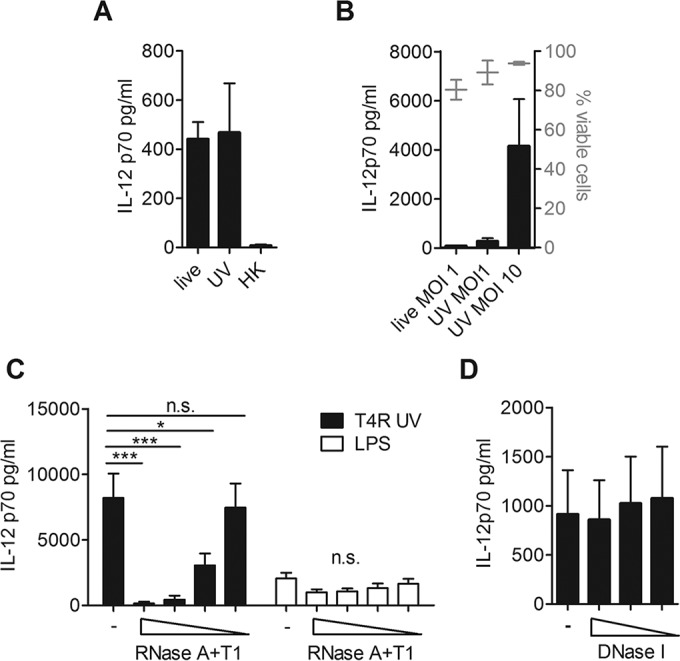
RNA is required as a pneumococcal stimulus to induce IL-12p70 production. DCs were challenged with live, UV-killed, or heat-killed (HK) T4R (A), with live or UV-killed T4R at the indicated MOI (B), with UV-killed T4R (MOI, 10), with LPS pretreated with a cocktail of RNase A (200 to 20 U/ml) and RNase T1 (8,000 to 800 U/ml) (C), or with UV-killed T4R (MOI, 10) pretreated with DNase I (1,000 to 250 U/ml) (D). IL-12p70 production in the cell supernatant was measured in an ELISA (A to D), and DC viability was measured by flow cytometry (B). Values represent means ± standard errors of the means for results from 4 (A), 3 (B), 7 (C), or 4 (D) experiments. Statistical analysis was performed using a one-way analysis of variance and a Bonferroni posttest. *, *P* < 0.05; **, *P* < 0.005; ***, *P* < 0.0005. n.s., not significant.

To investigate the role of RNA during the infection of DCs with whole pneumococci, we treated T4R with UV radiation or heat. We found heat treatment, in contrast to UV treatment, abolished bacterial induction of IL-12p70 (Fig. 6A). Treatment with UV preserved bacterial RNA, whereas the RNA was degraded during heat killing (see Fig. S3A in the supplemental material). Heat, in contrast to UV treatment, is also expected to melt double-stranded regions in RNA. UV-killed T4R triggered IL-12p70 production in amounts comparable to those produced via live T4R, and a 10-fold increase in the MOI of UV-killed bacteria increased cytokine secretion without affecting the viability of DCs (Fig. 6B). Similar to live bacteria, the IL-12p70 production in response to UV-killed bacteria was dependent on bacterial uptake (see Fig. S3B in supplemental material).

To study whether depletion of RNA from whole bacteria could decrease IL-12p70 production, we next treated UV-killed T4R with the cocktail of RNase A and RNase T1 prior to DC challenge. We used UV-killed bacteria for the RNA digestion, since the enzymatic treatment induced death in live bacteria (data not shown), which also could result in reduced cytokine production by DCs. The RNase treatment of UV-killed T4R led to a significantly reduced cytokine secretion by DCs in a dose-dependent manner, and the effect was specific for degradation of RNA, since pretreatment of LPS with the endonucleases did not affect cytokine secretion (Fig. 6C). In contrast, treatment of UV-killed T4R with DNase I did not affect IL-12p70 production (Fig. 6D). Hence, pneumococci equipped with intact RNA activate IL-12p70 production in DCs. Collectively, our results demonstrate that pneumococcal RNA is a stimulus for the TLR3/TRIF pathway, which is required to induce the production of IL-12p70 by DCs during infection with pneumococci, a pathway that can be primed by IAV coinfection.

## DISCUSSION

**FIG 7:**
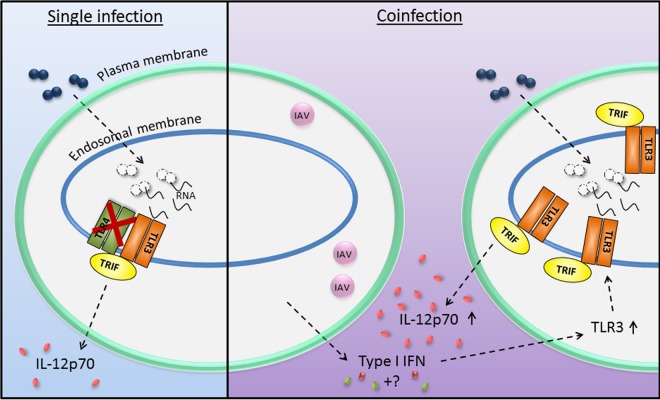
Proposed model of mechanisms involved in human DC responses to pneumococci or to coinfections with IAV and pneumococci. In single infections, pneumococci are taken up by DCs into the endolysosomal compartment. The pneumococcal RNA activates TLR3 and its adaptor molecule TRIF, which subsequently leads to IL-12p70 secretion by the DCs. This IL-12p70 secretion is independent of TLR4. In coinfections with IAV and pneumococci, IAV infects a subset of DCs and induces the secretion of type I interferons and other soluble factors (marked by a question mark in the figure). These soluble factors induce the surrounding DCs to express increased amounts of TLR3. The increased TLR3 expression in the primed DCs contributes to more efficient detection of pneumococci and to increased secretion of IL-12p70.

Within recent years, bacterial RNA has emerged as an important trigger of host immune responses to sense live bacteria (36), in both TLR3-independent (36–42) and TLR3-dependent manners (43–45). Clearly, the traditional view of TLR signaling and TLR3 as a sensor of viral dsRNA has to be reconsidered. Here, we describe for the first time a TLR4-independent and TLR3/TRIF-dependent IL-12 production in response to pneumococci, and we propose that the activation of TLR3 plays an important role in coinfections of DCs with IAV and pneumococci. The investigated aspects of DC responses to pneumococci and IAV, as well as the proposed mechanisms involved, are summarized in Fig. 7.

The role of pneumolysin as a TLR4 agonist is still being debated in the literature. Depending on the model system and cell type, it has been reported that pneumolysin activates TLR4 and induces the production of cytokines (12–16), whereas other studies show TLR4-independent cytokine production (46–48). Our results demonstrated that IL-12p70 production in human monocyte-derived DCs is independent of cellular expression of TLR4. Furthermore, we showed that bacterial uptake by human DCs is required for IL-12p70 production, as well as the expression of the endosomal receptor TLR3 and its adaptor molecule TRIF. The TLR3 dependence for IL-12p70 production is independent of capsular expression of the bacteria.

In murine-derived DCs infected with T4R, we previously found a much smaller effect on IL-12p70 secretion than in T4R-infected human DCs (35). Additionally, a redundancy of TLRs *in vivo* has also been found in mice in relation to pneumococcal infections for the receptors TLR1, TLR2, TLR4, and TLR6 (17, 49, 50). In these studies, only TLR9 was identified as a nonredundant receptor in a murine pneumococcal pneumonia model (17). Monocyte-derived human DCs do not express TLR9 (reviewed in reference 51), and the effects that we find *in vitro* might therefore be partially masked in murine *in vivo* models by the activation of other cell types and other TLRs, especially TLR9, explaining why we see only modest differences in infected TLR3 deficient mice as compared to wild-type mice (data not shown).

Here, we also identified a stimulus for IL-12p70 production by human DCs *in vitro*. The presence of bacterial RNA was required and sufficient to induce IL-12 responses, and we found that completely digested pneumococcal RNA lost its capacity to induce IL-12p70 secretion. Moreover, we demonstrated that pneumococcal RNA activates TLR3.

We propose that the TLR3-dependent induction of IL-12p70 is of special importance in coinfections with IAV, as severe bacterial diseases, and in particular those caused by pneumococci, often occur 1 to 2 weeks after the onset of IAV infections (5). We previously showed that IAV-infected DCs express type I IFNs, which prime the surrounding DCs to secrete increased amounts of IL-12p70 upon subsequent challenge with pneumococci (33). Viral infection, as well as type I IFNs or PolyI:C, are known to upregulate TLR3 expression (52–55). The role of TLR3 in IAV infections has been described elsewhere (54), as has the importance of TLR3 for the priming which leads to severe secondary infections with *S. pneumoniae* (56), but so far the contribution of TLR3 during the secondary infection with pneumococci has not been studied. Here, we show *in vitro* that IAV upregulates TLR3 expression in DCs and that the enhanced IL-12p70 production in coinfected cells depends on TLR3. We attribute only a partial role in the upregulation of TLR3 to type I IFNs, since IFN-α alone has no impressive effect on the upregulation of TLR3 in DCs. Therefore, there are likely additional soluble factors involved in the priming of human DCs by IAV.

In summary, we propose a model in which pneumococci are phagocytosed by DCs and degraded in the endolysosomal compartment. The released pneumococcal RNA activates TLR3 and TRIF, which subsequently leads to IL-12 expression and secretion. In the coinfection setting, IAV primes DCs to secrete type I IFNs and other soluble factors, which trigger changes in the surrounding DCs, including enhanced expression of TLR3, which prime the cells to react with increased IL-12p70 production in the secondary infection with pneumococci (Fig. 7).

## MATERIALS AND METHODS

### Bacterial and virus strains used.

The encapsulated serotype 4 strain TIGR4 (T4; ATCC BAA-334) (57) of *S. pneumoniae* was used, as well as the unencapsulated isogenic mutant T4R (58). Bacteria were grown on blood agar plates at 37°C and 5% CO_2_ overnight. Colonies were inoculated into C+Y medium and grown until exponential phase (optical density at 620 nm [OD_620_], 0.5). Dilutions were made to obtain the desired concentration of bacteria, and viable counts were performed to retrospectively confirm the bacterial numbers. The X31 strain of IAV (59) was originally propagated in chicken eggs, purified, and concentrated on a sucrose gradient. The virus was further propagated for one generation in Madine-Darby canine kidney (MDCK) cells, purified, and concentrated on a sucrose gradient (Virapur). Virus titers were determined by performing Avicel (FMC Bioploymer) plaque assays on MDCK cells as described elsewhere (60).

### Culturing of human DCs.

Monocytes were purified from buffy coats of healthy donors (Karolinska University Hospital) by using a RosetteSep monocyte purification kit (Stem Cell Technologies) and Ficoll-Hypaque Plus (Amersham Biosciences) gradient centrifugation. Human DCs were seeded at 0.8 × 10^6^ to 1.5 × 10^6^ cells/ml in R10 (RPMI 1640, 2 mM l-glutamine, 10% fetal bovine serum [FBS]) supplemented with granulocyte-macrophage colony-stimulating factor (GM-CSF; 40 ng/ml) and IL-4 (40 ng/ml) (both from PeproTech) for 6 days. Cells were given fresh media and cytokines at a ratio of 1:1 on day 4 and cultured until day 6. The human DC phenotype was assessed by examination of CD11c and CD1a expression via staining with allophycocyanin (APC)-conjugated mouse anti-human CD11c and fluorescein isothiocyanate (FITC)-conjugated mouse anti-human CD1a (BD Pharmingen). DCs used in these experiments were above 90% CD1a^+^/CD11c^+^.

### Inhibitors and reagents.

LPS, PolyI:C, R848, cytochalasin D, and wortmannin were purchased from Sigma, and the TLR3/dsRNA complex inhibitor was purchased from Roche. Human type I IFN-α was purchased from PBL Assay Science. Cytochalasin D (0.5 mM), wortmannin (0.1 mM), and the TLR3/dsRNA complex inhibitor (270 µM) were applied 20 min prior to infection. LPS (100 ng/ml), PolyI:C (10 µg/ml), R848 (10 µg/ml), and IFN-α (500 U/ml) were used for stimulation of TLR4, TLR3, TLR7/8, and IFNAR, respectively.

### siRNA knockdown.

DCs (6 × 10^6^) were electroporated with 5 µM siRNA against TRIF (s45113, s45114, and s45115), TLR4 (s14194, s14195, and s14196), TLR3 (s235 and s236), or random control siRNA (4390843 and 4390846) (all from Life Technologies) on day 4 of DC differentiation. The cells were electroporated with the Bio-Rad Gene Pulser (square wave, 500 V, 0.5 ms with a single pulse), immediately resuspended in fresh culture medium containing IL-4 and GM-CSF, and incubated for a further 2 days.

### *In vitro* infection of DCs.

DCs were seeded in 96-well plates (1 × 10^5^ per well) and exposed to pneumococci. If not otherwise stated, an MOI of 1 was used, and extracellular bacteria were killed with 200 µg/ml gentamicin after 2 h of infection and maintained in culture until the experiment was ended after 18 h. For opsonization, bacteria were incubated in the presence of pneumococcal antiserum for serotype 4 from Statens Serum Institut for the duration of the uptake period. For coinfection experiments, DCs were exposed to IAV at a MOI of 1 under serum-free conditions for 1 h and in the presence of serum for 3 h. Cells were pelleted, medium was removed, and T4R was added in fresh R10 medium. After a 2-h incubation, extracellular bacteria were killed with 200 µg/ml gentamicin and maintained in the cell culture until the experiment was ended after 18 h.

### Assessment of cell viability.

The influence of pneumococcal infection on DC viability was determined using annexin V-FITC (BD Pharmingen) and the fixable viability dye (FVD) eFluor780 (eBioscience). Cells were infected as previously described, and 18 h after infection the numbers of apoptotic and necrotic cells were determined by staining cells with annexin V-FITC and the FVD eFluor780 in Annexin V buffer and fixation with 2% paraformaldehyde. Cells were assessed by flow cytometry in a Gallios flow cytometer.

### RNA isolation, cDNA synthesis, and quantitative RT-PCR.

Total cellular RNA was extracted from infected cells using the RNeasy kit (Qiagen). The concentration and purity of isolated RNA were determined spectrophotometrically with the NanoDrop ND 1000 apparatus. cDNA was synthesized from the isolated RNA using the High Capacity cDNA reverse transcription kit (Applied Biosystems). RT-PCR was performed using the iTaq Universal SYBR green supermix (BioRad). Predesigned primer mixes containing forward and reverse primers for the specific RT-PCR target were purchased from Qiagen (QuantiTect primer assay). The following primers were used: TRIF, Hs_TICAM1_1_SG; TLR4, Hs_TLR4_2_SG; TLR3, Hs_TLR3_1_SG; IL-12p40, Hs_IL12B_1_SG; glyceraldehyde-3-phosphate dehydrogenase (GAPDH), Hs_GAPDH_1_SG. Each primer pair was validated for specificity by performing melt curve analysis of the PCR product to ensure the absence of primer dimers and unspecific products. For each sample, the mRNA expression level was normalized to the level of GAPDH, and relative expression was determined via the ΔΔ*C_T_* method. Each PCR run included no-template controls.

### Quantification of cytokines.

For cytokine assessment *in vitro*, culture supernatants were harvested 18 h after infection and frozen at −20°C or used directly for measurement of IL-12p70 by using the OptEIA enzyme-linked immunosorbent assay (ELISA) kit (BD Biosciences).

### Pretreatments of bacteria with UV, heat, or enzymatic digestion.

Bacteria were killed by incubation at 95°C for 20 min or by exposure to 1,200 J/m^2^ UV radiation for 20 min. Death was confirmed by plating aliquots of the bacterial suspensions on blood agar plates and incubating at 37°C and 5% CO_2_ overnight.

UV-killed bacteria were incubated with a cocktail of RNase A (200 to 20 U/ml) and RNase T1 (8,000 to 800 U/ml) (Life Technologies) or with DNase I (1,000 to 250 U/ml; Qiagen) in a volume of 50 µl at 37°C for 1 h prior to infection of DCs. Total bacterial RNA was incubated with 25 U/ml RNase A and 1,000 U/ml RNase T1 of the RNase cocktail in a volume of 25 µl for 30 min.

### RNA transfection.

T4R was grown to mid-log phase and treated with phenol. Total RNA was isolated with TRIzol (Life Technologies) followed by DNA digestion with Turbo DNase (Life Technologies). RNA concentration and quality were determined spectrophotometrically with the NanoDrop ND 1000 apparatus, and the absence of DNA contamination was confirmed by PCR. DCs were transfected with 1 µg bacterial RNA per well complexed with *N*-[1-(2,3-dioleoyloxy)propyl]-*N*,*N*,*N*-trimethylammonium methylsulfate (DOTAP; Roche) at a ratio of 1:2.5 and incubated for 18 h. HEK293 cells were transfected with 1 µg RNA per well complexed with Lipofectamine 2000 (Life Technologies) at a ratio of 1:2 and incubated for 6 h.

### Luciferase assay with transfected HEK293 cells.

HEK293 cells stably expressed human TLR4, MD2, and CD14 as well as luciferase under the control of the NF-κB promoter or human TLR3 as well as luciferase under the control of the ELAM promoter (61, 62). The cells were cultured in Dulbecco’s modified Eagle’s medium supplemented with 10% FBS and penicillin-streptomycin. A total of 2.5 × 10^4^ cells were seeded per well of a 96-well plate. After 1 day, the cells were transfected with 1 µg pneumococcal RNA and incubated for 6 h. Cells were lysed, and luciferase activity was measured using the luciferase assay system (Promega) according to the manufacturer’s instructions.

### Visualization of RNA on gels.

Isolated total RNA was loaded on a RNA nanochip and analyzed and visualized with the Agilent 2100 Bioanalyzer.

### Statistical analysis.

Data were statistically analyzed as indicated in the figure legends, using GraphPad Prism 5.04. Significant differences were noted at *P* levels of <0.05, <0.005, and <0.0005.

## SUPPLEMENTAL MATERIAL

Figure S1  Infection of DCs with T4R shows uptake-dependent IL-12p70 production. DCs were infected with T4R, and IL-12p70 was measured in the cell supernatant. DC viability was measured by flow cytometry (A). Uptake of bacteria by DCs was inhibited with cytochalasin D and wortmannin (B). Values represent means ± standard errors of the means for results from 4 (A) or 5 (B) experiments. cytD, cytochalasin D; WM, wortmannin. Download Figure S1, TIF file, 0.9 MB

Figure S2  Enhanced IL-12p70 production in DCs primed with IFN-α requires TLR3. DCs were treated with IFN-α for 4 h, and expression of TLR3 was measured by RT-PCR after 8 h (A). DCs were primed with IFN-α for 4 h and/or subsequently infected with T4R with or without a TLR3 inhibitor, as indicated (B). DCs were primed with IAV for 4 h and/or subsequently transfected with total RNA from T4R with or without a TLR3 inhibitor, as indicated (C). IL-12p70 secretion was measured in the supernatant by ELISA (B and C). Values represent means ± standard errors of the means for results from 3 (A), 6 (B), or 5 (C) experiments. Statistical analysis was performed using Student’s *t* test (RT-PCR) or a Wilcoxon matched-pairs signed-rank test (ELISA). *, *P* < 0.05. n.s., not significant. Download Figure S2, TIF file, 1.2 MB

Figure S3  UV-killed pneumococci induce IL-12p70 production similar to live pneumococci. Total RNA was isolated from live T4R or UV-killed or heat-killed T4R and visualized by gel electrophoresis on an RNA nanochip (A). DCs pretreated with cytochalasin D and wortmannin were challenged with UV-killed T4R (MOI, 10), and IL-12p70 production was measured in the cell supernatant (B). Graph shows the means ± standard errors of the means for results from 4 experiments. HK, heat killed; cytD, cytochalasin D; WM, wortmannin. Download Figure S3, TIF file, 0.6 MB
