# Supplementary figures and images for: Toll-Like Receptor 3/TRIF-Dependent IL-12p70 Secretion Mediated by Streptococcus pneumoniae RNA and Its Priming by Influenza A Virus Coinfection in Human Dendritic Cells
Source: mBio. 2016 Mar 8;7(2):e00168-16. doi: 10.1128/mBio.00168-16 (PMC4810485; doi:10.1128/mBio.00168-16)

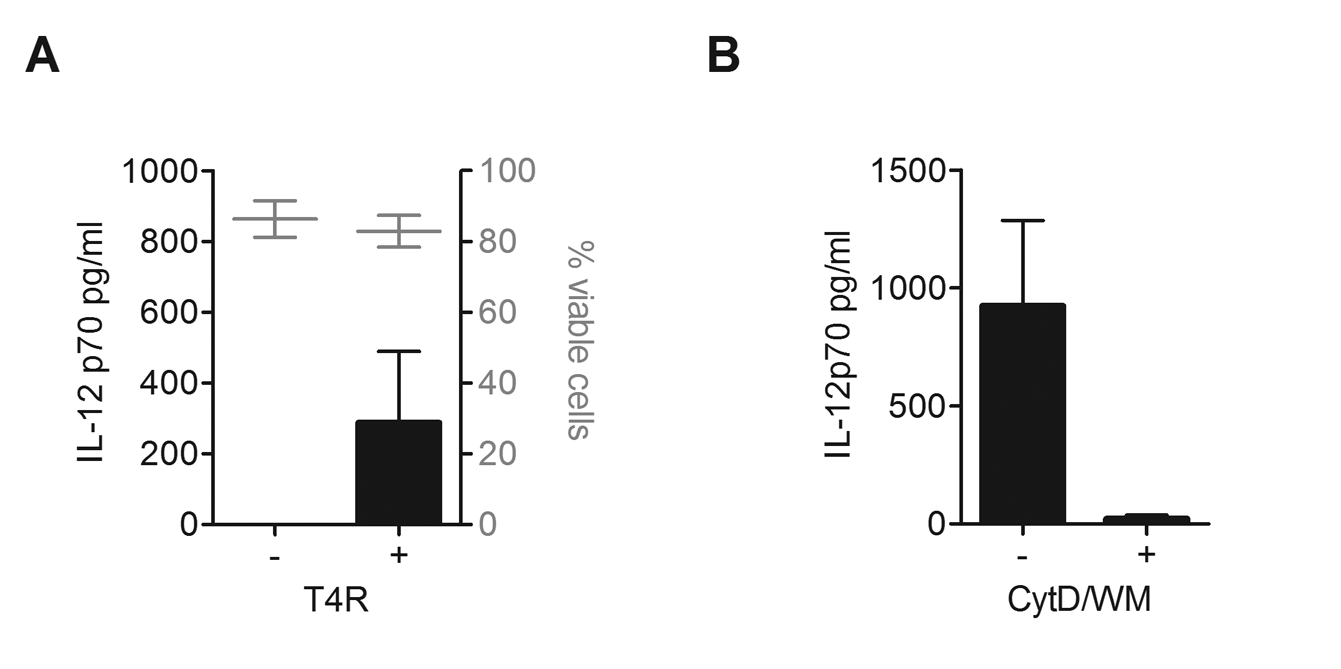

Supplement: Figure S1 — Infection of DCs with T4R shows uptake-dependent IL-12p70 production. DCs were infected with T4R, and IL-12p70 was measured in the cell supernatant. DC viability was measured by flow cytometry (A). Uptake of bacteria by DCs was inhibited with cytochalasin D and wortmannin (B). Values represent means ± standard errors of the means for results from 4 (A) or 5 (B) experiments. cytD, cytochalasin D; WM, wortmannin. Download [file mbo001162719sf1.tif]

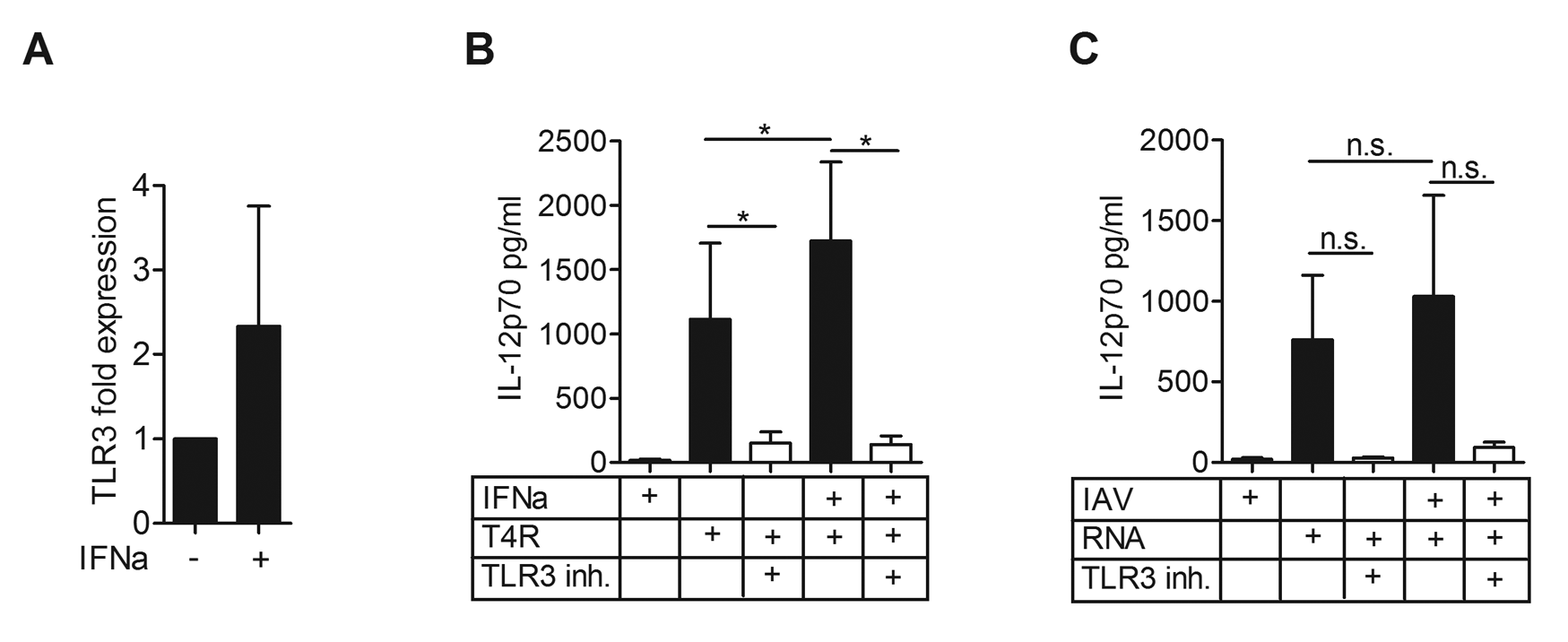

Supplement: Figure S2 — Enhanced IL-12p70 production in DCs primed with IFN-α requires TLR3. DCs were treated with IFN-α for 4 h, and expression of TLR3 was measured by RT-PCR after 8 h (A). DCs were primed with IFN-α for 4 h and/or subsequently infected with T4R with or without a TLR3 inhibitor, as indicated (B). DCs were primed with IAV for 4 h and/or subsequently transfected with total RNA from T4R with or without a TLR3 inhibitor, as indicated (C). IL-12p70 secretion was measured in the supernatant by ELISA (B and C). Values represent means ± standard errors of the means for results from 3 (A), 6 (B), or 5 (C) experiments. Statistical analysis was performed using Student’s t test (RT-PCR) or a Wilcoxon matched-pairs signed-rank test (ELISA). *, P < 0.05. n.s., not significant. Download [file mbo001162719sf2.tif]

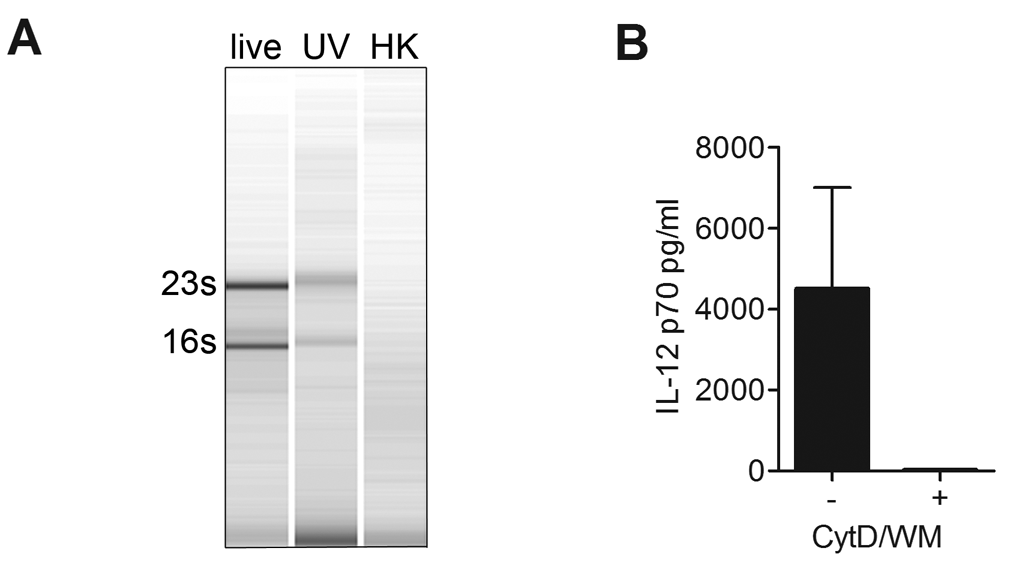

Supplement: Figure S3 — UV-killed pneumococci induce IL-12p70 production similar to live pneumococci. Total RNA was isolated from live T4R or UV-killed or heat-killed T4R and visualized by gel electrophoresis on an RNA nanochip (A). DCs pretreated with cytochalasin D and wortmannin were challenged with UV-killed T4R (MOI, 10), and IL-12p70 production was measured in the cell supernatant (B). Graph shows the means ± standard errors of the means for results from 4 experiments. HK, heat killed; cytD, cytochalasin D; WM, wortmannin. Download [file mbo001162719sf3.tif]
